# Impact of Protocol Amendments, Personnel Experience and Social Determinants of Health on Study Protocol Adherence in Clinical Trials with Combination Products

**DOI:** 10.1007/s43441-025-00859-y

**Published:** 2025-08-16

**Authors:** K. N. Cilley, A. O. Kaliaev, M. A. Malikova

**Affiliations:** 1 Department of Surgery, Boston University, Chobanian and Avedisian School of Medicine, Boston Medical Center, 85 East Concord Street, 02118 Boston, MA United States; 2 Department of Radiology, Boston Medical Center, Boston, United States

**Keywords:** Good clinical practice (GCP), Protocol deviations, Combination products, Risk-based approach, Protocol amendments, Social determinants of health, Study protocol complexity, Protocol adherence, Data integrity, Study staff experience

## Abstract

**Introduction:**

The amendments to the International Council for Harmonization (ICH) Good Clinical Practice (GCP) E8 guidelines were introduced to enhance clinical trial quality, patient safety, and efficiency through a more patient-centric, risk-based approach. This study investigates the impact of various study risk factors such as protocol amendments, informed consent changes, protocol complexity, and social determinants of health (SDOH) on protocol deviations and patient retention in clinical trials involving combination products.

**Methods:**

A retrospective analysis of 14 clinical trials with 202 enrolled subjects was conducted. Key risk indicators (KRIs) such as protocol amendments, amendments triggering informed consent changes, study staff experience, and clinical trial phase were evaluated for their association with protocol deviations. The analysis also explored the influence of social factors, including age, gender, race, insurance type, and travel distance on protocol adherence.

**Results:**

Study revealed that longer study participation was associated with an increased number of protocol deviations (*p* = 0.0003), while no significant associations were found between protocol deviations and demographic factors (*p* = 0.4039 for gender; *p* = 0.40650 for age), insurance type (*p* = 0.0640), or complexity scores (*p* = 0.7798). The findings highlight the importance of effective informed consent processes, study staff training, and risk management strategies to minimize protocol deviations and enhance data integrity in clinical trials.

**Conclusion:**

while larger numbers of participant were associated with more deviations, site preparedness and patient compliance can mitigate these risks, underscoring the need for robust quality management systems in clinical trials.

## Introduction

The International Council for Harmonization (ICH) Good Clinical Practice (GCP) E8 amendments were introduced to align with modern clinical trial practices and improve clinical trial quality, safety, and efficiency globally [1]. The key amendments to ICH E8, effective in April of 2022, emphasized patient-centricity and risk-based approaches with a focus on safety and well-being throughout the clinical trial process [1]. There was also an emphasis placed on informed consent that includes patient needs, preferences, and cultural contexts to ensure patients understand the clinical trial and its risks. In the revised regulatory guidance minimizing patient burden was also prioritized by request to make the trial designs and study protocols more adaptable and patient friendly [2]. Also, a shift toward a risk-based approach to quality management was introduced. This means identifying and mitigating risks in clinical trials proactively and upfront at the study protocol design stage, rather than relying on extensive monitoring in the execution phase. Sponsors and investigators are encouraged to proactively assess risks to trial integrity, patient safety, and data quality and apply appropriate resources to mitigate these risks.

The amended ICH E8 guidance highlighted data quality throughout the trial process and underscored the importance of ensuring data are both reliable and accurate, regardless of the methods used for data collection (e.g., digital tools, remote monitoring). The guidelines also outlined monitoring and data management strategies to ensure consistent and reliable data, including flexible monitoring strategies, as opposed to traditional, on-site visits. In addition, the amendments stressed the importance of establishing a robust quality management system for trials to ensure compliance with GCP and good documentation practices. Risk-based monitoring should be incorporated into the trial’s overall quality management system, ensuring resources are focused on areas with the highest risk [1]. 

There is a stronger focus on ensuring diverse populations in clinical trials, to better reflect the populations that will use the medical products once they are marketed [3–5]. This is essential for improving the generalizability of trial results. In order to achieve equity in trial design the following factors at minimum should be considered: age, gender, race, primary spoken language, health insurance status to prevent health disparities in the treatment of different groups. Diversity in clinical trial populations and inclusive practices are key to improving trial outcomes and generalizability [3–5]. Overall, these amendments aim to enhance the quality and efficiency of clinical trials, prioritize patient-centric approaches, and support the innovative use of technologies in clinical research.

According to Getz K., et at., a recent trend in clinical trials is an increase in the number of amendments to study protocols and the time required for these amendments to be enforced [6]. Because each investigational site has a different time frame for obtaining amendment approval from centralized or local institutional review boards (IRBs), often multiple protocol versions are being implemented simultaneously across different study sites [6]. Also, many protocol amendments trigger informed consent changes, leading to further discrepancies and delays between sites.

In addition to an increase in the number of protocol amendments, studies have shown a rise in protocol complexity, which may impact protocol adherence, data integrity, and the overall quality of clinical trials [7]. As described previously by Malikova M.A., protocol’s complexity score is determined by various factors, including the eligibility criteria, product administration, number of study groups, and length of the treatment phase. This score can give study staff insight into how difficult a protocol is to adhere to and help to develop risk mitigation strategies to improve study protocol adherence during the execution phase [8]. 

Protocol deviations occur when study staff or participants fail to meet the requirements outlined in the protocol, and they can be used to measure how closely patients and investigational sites are following study guidelines [9]. While high numbers and rates of protocol deviations mainly pose a risk to data validity, protocol deviations can also affect patient confidentiality and safety [9]. Additionally, patients who drop out or are withdrawn from studies before their projected end date have the potential to impact the quality of clinical trial data [10]. 

As emphasized previously by Kaliaev A.O and Malikova M. A., and Ting J, et al., due to complex nature of combination products, which consist of different mixture of drugs, devices, and biologics, have more complex regulatory, storage, and application guidelines than their constituent parts alone, they also have the potential to affect protocol deviation rates in the studies in which they are used [11, 12]. 

The purpose of this project was to investigate how various study risk factors, such as the number of protocol amendments, amendments triggering informed consent changes, and protocol complexity scores, correlate with protocol deviations in clinical trials with combination products. We also evaluated whether the number and experience levels of study staff, clinical trial phase, the primary mode of action (PMOA), and social determinants of health (SDOH), affect the protocol deviations and data quality and integrity.

## Methods

A retrospective analysis of 14 clinical trials with 202 enrolled subjects, involving investigational combination products, was conducted to evaluate the relationship between protocol deviations and critical quality key risk indicators (KRIs) (e.g. protocol amendments, consent form changes, numbers and experience levels of study staff, and lengths of the treatment and follow-up phases). The correlation of study protocol deviations with social determinants of health (e.g. race, gender, age, insurance type, travel distance, income, and primary language, etc.) was assessed.

Travel distance was calculated using the distance between the investigational site clinic and the enrolled subject’s home address. Because some subjects did not provide their home addresses, their travel distance was calculated using the distance from the investigational site to their recorded zip code. Census data was used to obtain median household income using each patient’s zip code and year of enrollment [13]. Insurance types were recorded as one of three categories: private, government, or no insurance. Patients were split into 3 body mass index (BMI) groups, including BMI < 25, BMI = 25-29.99, and BMI = 30+. They were also divided into two groups for obesity status, which were BMI < 30 for “not obese” and BMI = 30 + for “obese” [14]. Complexity scores were determined using the methodology described previously by Malikova M.A [15]. 

The number of protocol deviations was assessed to monitor protocol adherence within the 14 examined clinical trials. The rate of protocol deviations, which was calculated by dividing the number of protocol deviations in each trial by the number of patients enrolled in that trial, was also assessed. Protocol deviations were broken down into the following seven categories: study visit out of window, study visit missed, use of prohibited concomitant medication, test article handling, failure to perform a study procedure or study procedure performed late, eligibility criteria not met, and “other”. The length of study participation was determined for each study by calculating the number of days from the date the subject signed the consent form to the date the subject completed the trial.

The number of protocol amendments was recorded, along with the number of amendments that triggered a change in the informed consent form, to determine how often study staff had to alter study procedures, timelines, or informed consent processes throughout a study. The number of study coordinators and investigators on each trial was also recorded to determine whether a greater number of study team members and personnel changes introduced more variability and affected the number of protocol deviations. The experience of each coordinator and investigator was calculated using the time in months between their first research position or their first ever research publication and the date they were added to the study of interest (e.g. signed the delegation of authority log or were added to the study with local or central Institutional Review Board (IRB)). The primary mode of action (device, drug, or biologic) and the clinical trial phase were recorded for each trial to assess the effect of each on protocol adherence.

The number of protocol deviations was compared to various social determinants of health (SDOH) using the distribution of Wilcoxon scores from Kruskal-Wallis tests. Also, the Spearman correlation test was used to assess associations between examined variables. The Spearman correlation coefficient of 0 represents no correlation, while values close to -1 or + 1 indicate strong correlations. A negative correlation coefficient indicates an inverse relationship between the two variables and a positive coefficient represents a direct relationship [16]. The strength of the association can be further broken down into the following categories: r = 0.1–0.3 weak, 0.4–0.6 moderate, and 0.7–0.9 strong [16]. The total number and rate of protocol deviations in each trial were compared to the number of protocol amendments, amendments that triggered consent form changes, the number and experience of study staff, the primary mode of action, and the trial phase using Kendall’s tau correlation. Kendall’s tau test was used for these variables due to data limitations that did not meet the requirements for Spearman’s correlation test [17]. Kendall’s tau coefficient (after the Greek letter ”τ”, tau), also known as the Kendall rank correlation coefficient, is a statistic used to measure the ordinal association between two measured quantities. It is a non-parametric measure of relationships between columns of ranked data, returning a value between 0 and 1, where 0 indicates no relationship and 1 indicates a strong relationship [17]. 

## Results

Retrospective analysis of 14 combination product clinical trials, with 202 enrolled patients summarized in Table 1. According to the primary mode of action, 50% of the combination products within the 14 trials were classified as a device, 7.1% were classified as a drug, and 42.9% were classified as a biologic. 9 (64.3%) of the trials were phase 3, 3 (21.4%) were phase 4, and 2 trials (14.3%) were phase 2. The total number and rates of protocol deviations observed in each trial were also recorded as summarized in Table 1.

**Table 1:** Summary of studies with combination products by regulatory designation, primary mode of action, and study protocol deviations. *Abbreviations*: VLU-venous leg ulcer, DFU- diabetic foot ulcer, SUG- surgical study. PMOA- primary mode of action, FDA- food and drug administration, IDE- investigational device exemption, IND- investigational new drug application, PD- protocol deviations, PAD- peripheral arterial disease, CLI- critical limb ischemia. PTFE- polytetrafluoroethylene graft for vascular surgery

Study Name	Phase	Indication	PMOA	FDA Designation (510 K, IDE, IND)	Combination Type	Total Number of Subjects Enrolled	Total Number of PDs	Rate of PDs (Total #PDs/ Total Enrolled) per study
DFU-1	4	DFU	Device	510k	Device + biologic	6	13	2.17
DFU-2	3	DFU	Device	510k	Device + biologic	21	81	3.86
DFU-3	3	DFU	Device	IDE	Device + drug	1	9	9
DFU-4	3	DFU	Drug	IND	Drug + device	5	17	3.4
VLU-1	3	VLU	Biologic	IND	Biologic + biologic	24	119	4.96
VLU-2	3	VLU	Device	510 K	Device + drug	3	4	1.33
VLU-3	3	VLU	Biologic	IND	Biologic + biologic	18	74	4.11
VLU-4	3	VLU	Biologic	IND	Biologic + biologic	11	10	0.91
VLU-5	3	VLU	Biologic	IND	Biologic + biologic	2	12	6
VLU-6	2	VLU	Biologic	IND	Biologic + biologic	15	44	2.93
VLU-7	2	VLU	Biologic	IND	Biologic + biologic	15	12	0.8
SUG-1	4	Surgical wound closure	Device	510 K	Device + device	9	20	2.22
SUG-2	4	PAD with CLI	Device	IDE	Device + drug	68	251	3.69
SUG-3	3	Control of anastomosis site bleeding in vascular reconstructive surgery with PTFE	Device	IDE	Device + drug	4	5	1.25

**Fig. 1:**
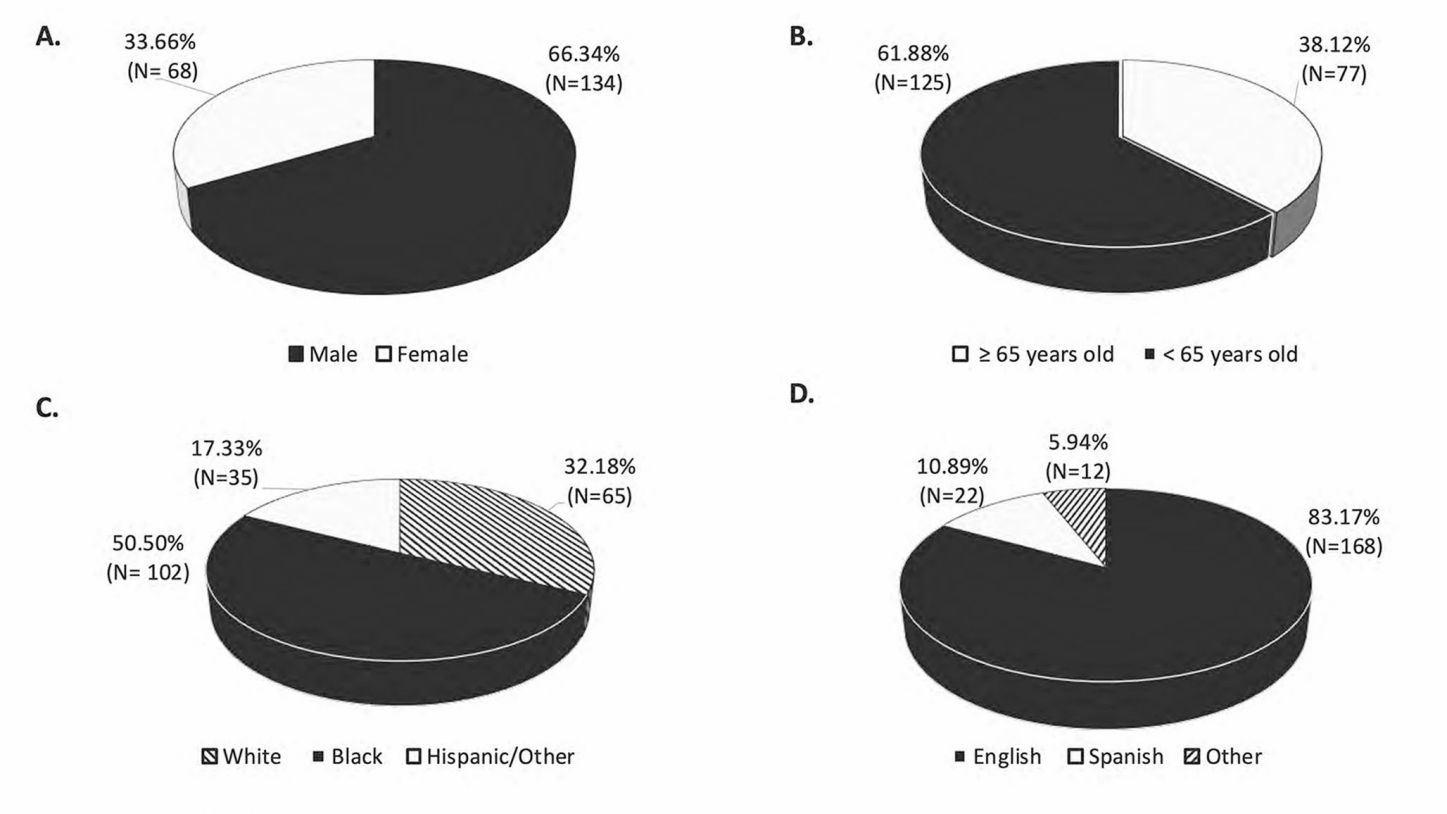
Study population demographic variables: (**A**) Study participants gender, (**B**) Study participants age, (**C**) race group (White, Black, other), (**D**) primary spoken language (English, Spanish, other)

**Fig. 2:**
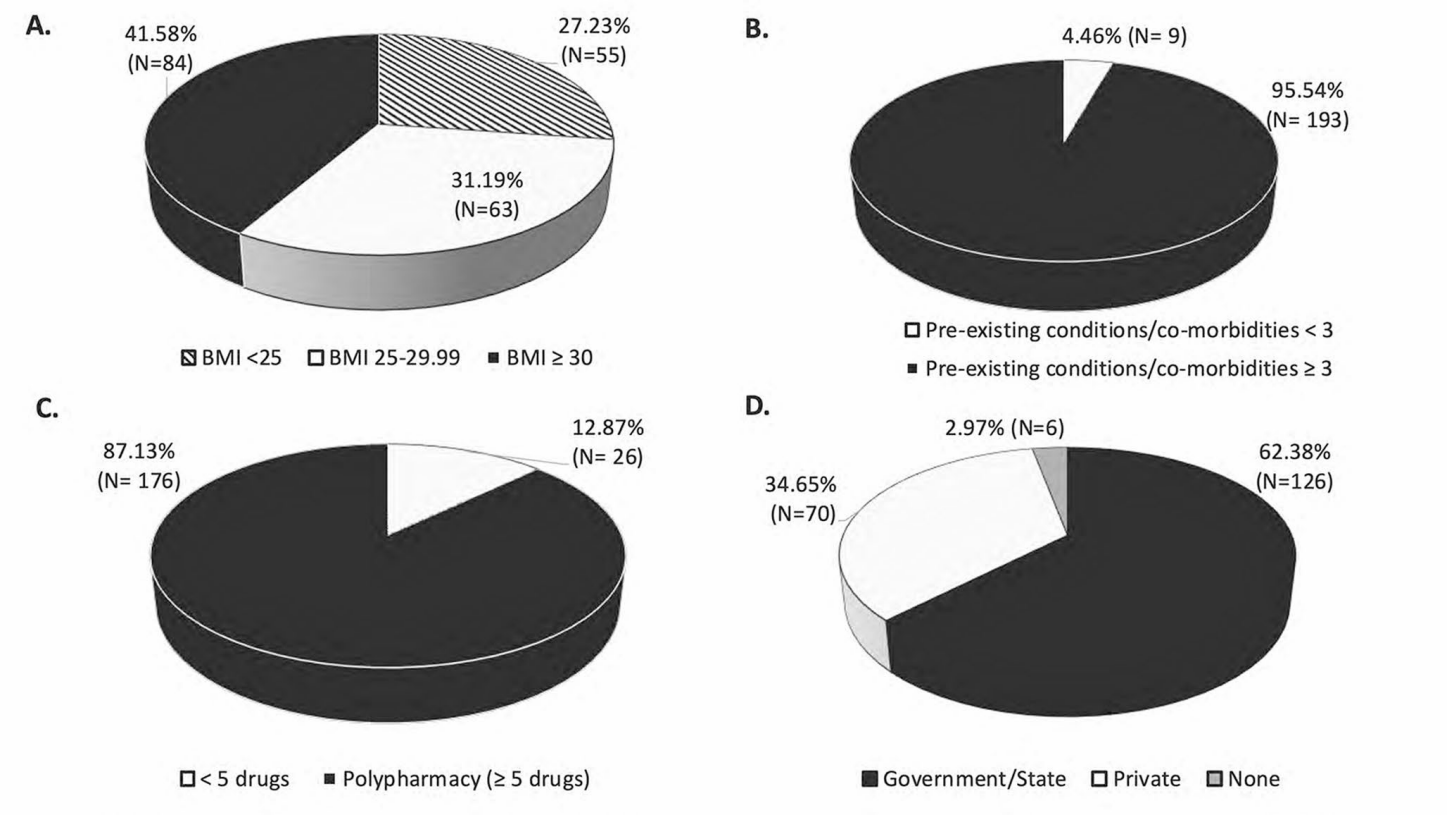
Study participants’ medical history and insurance status at the time of enrollment. (**A**) Study population BMI assessment (BMI < 25, 25-29.99, >30). (**B**) Number of participants’ pre-existing medical conditions (comorbidities). (**C**) Number of concomitant medications study participants were taking at the time of enrollment. (**D**) Type of medical insurance carrier

Of the 202 enrolled patients, 33.66% (*N* = 68) were females, 66.34% (*N* = 134) were males (Fig. 1A); and 61.88% (*N* = 125) were under 65 years old (Fig. 1B). As shown in Figs. 1C and 65 patients were white (32.18%), 102 were black (50.50%), and 35 belonged to another race category (17.33%). For primary spoken language, 83.17% spoke English (*N* = 168), 10.89% spoke Spanish (*N* = 22), and the remaining 5.94% (*N* = 12) spoke a different language. Additionally, 41.58% (*N* = 84) had a BMI of 30 or above, 31.19% (*N* = 63) had a BMI between 25 and 29.99, and 27.23% (*N* = 55) had a BMI of less than 25 as shown in Fig. 2A. Regarding co-morbidities and concomitant medications, 95.54% (*N* = 193) of patients had 3 or more pre-existing conditions, and 86.70% (*N* = 176) were taking five or more medications at the time of enrollment (Fig. 2B and C, respectively). Finally, in Fig. 2D the distribution of insurance type across all study patients is summarized, with 62.38% (*N* = 126) having government/state insurance carriers, 34.65% (*N* = 70) private insurance, and 2.97% (*N* = 6) having no insurance.

**Fig. 3:**
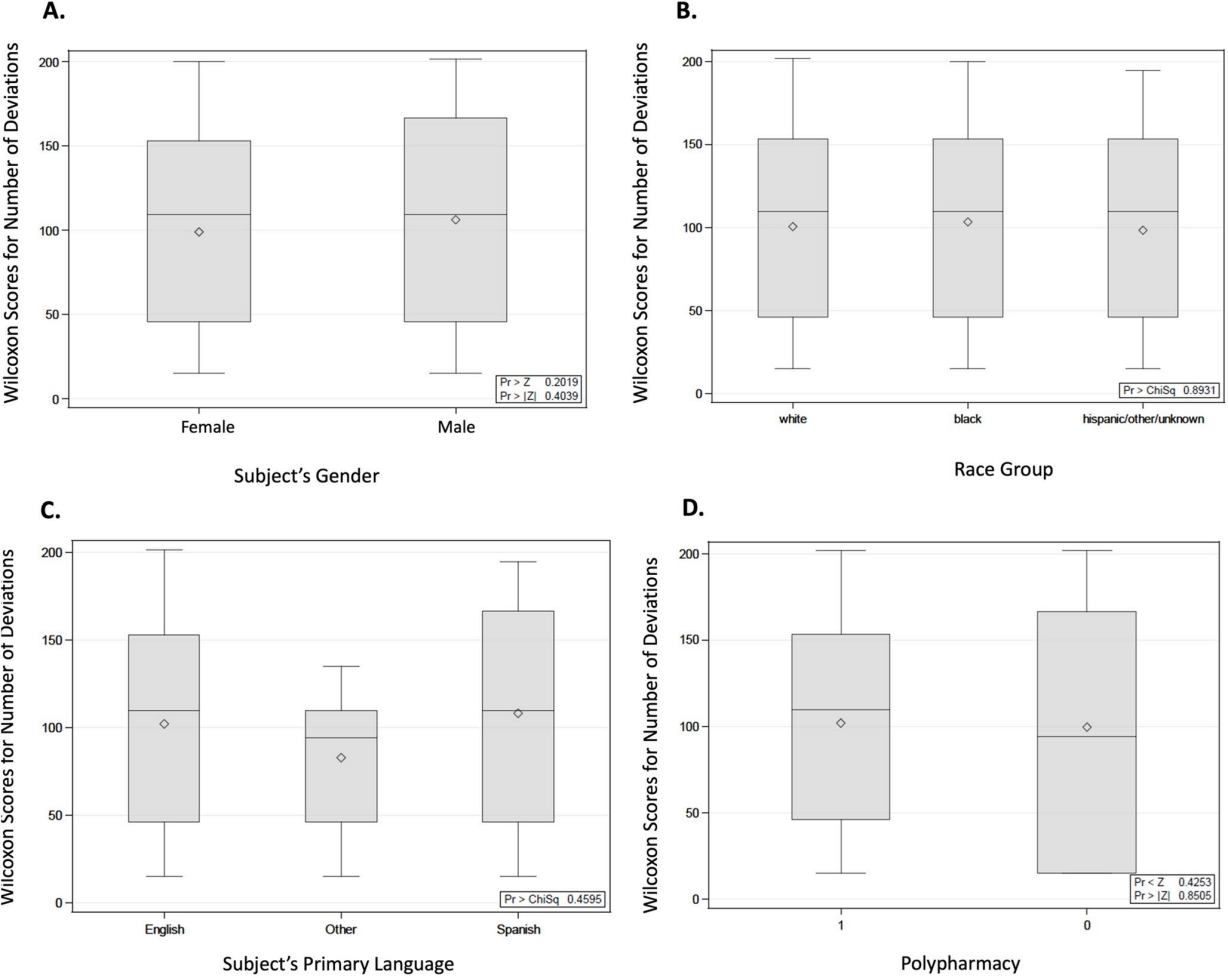
Wilcoxon score distributions for the number of study protocol deviations based on (**A**) gender; (**B**) race group; (**C**) primary language; (**D**) polypharmacy (number of concomitant medications at enrollment ≥ 5)

According to the Kruskal-Wallis test, there was no association found between gender and the number of protocol deviations (*p* = 0.4039) (Fig. 3A). Also, there was no correlation found between subject age and protocol deviations (*p* = 0.40650). Similarly, there was no association observed between race and protocol deviations (Fig. 3B), with a median value of deviations equal to 3 for all analyzed race groups (*p* = 0.8931). In addition, there was no association found between the distribution of primary languages and the number of protocol deviations, as shown in Fig. 3C (*p* = 0.4595).

Noteworthy, subjects that traveled farther distances to the clinic were not found to experience a higher number of protocol deviations (*p* = 0.5504). In terms of general health status assessment, a higher number of comorbidities was not associated with a higher number of protocol deviations (*p* = 0.1489), according to the Kruskal-Wallis test. Also, as summarized in Fig. 3D, there was no association found between polypharmacy (subjects that were taking ≥ 5 medications at the time of enrollment) and protocol deviations (*p* = 0.8505).

**Fig. 4:**
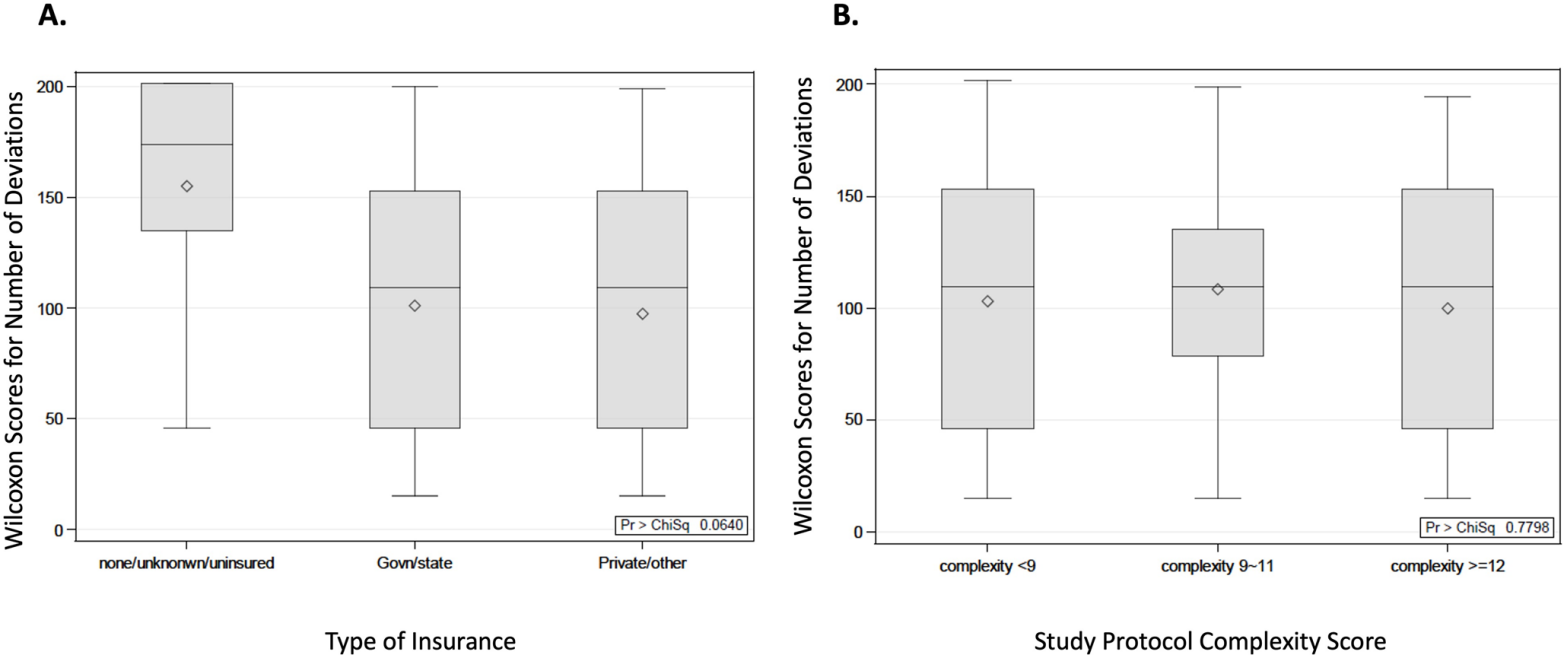
Wilcoxon score distributions for the number of study protocol deviations based on type of insurance (**A**) and study protocol complexity score (**B**)

Although the correlation was not statistically significant (*p* = 0.0640), there was a slight association found between insurance type and the number of protocol deviations, with a median value of 3 protocol deviations for both the government/state insurance and private/other insurance groups and a median value of 7 protocol deviations for the uninsured/unknown insurance group as highlighted in Fig. 4A. Utilizing the Kruskal-Wallis test, we did not find an association between complexity score and protocol deviations (*p* = 0.7798), as shown in Fig. 4B. Similarly, no association was observed between BMI and the number of protocol deviations (*p* = 0.2043), as well as between median household income and the number of protocol deviations (*p* = 0.2171).

There was an association found between the length of study participation and the number of protocol deviations and results were statistically significant (*p* = 0.0003). The Spearman correlation coefficient (r) for the observed relationship was 0.25318, indicating a weak correlation. Kendall-tau test showed that while there was an association (*p* = 0.0004) and strong correlation (τ = 0.72222) between a higher number of patients enrolled and a higher number of protocol deviations, no association was observed between a higher number of patients enrolled and a higher rate of protocol deviations (*p* = 0.9127). There was no association found between the study complexity score and the number of protocol deviations (*p* = 0.5376), or with the study complexity score and the rate of protocol deviations (*p* = 0.1793).

The Kendall-tau test also did not demonstrate an association between a higher number of protocol amendments and a higher number or rate of protocol deviations (*p* = 0.2034 and 0.3770, respectively). Similarly, no association was observed between the number of amendments that triggered an informed consent form (ICF) change and the number or rate of protocol deviations (*p* = 0.8124 and 0.4769, respectively). The Kendall-tau test did not find an association between coordinator experience and the total number of protocol deviations (*p* = 0.4414), or coordinator experience and the rate of protocol deviations (*p* = 0.1698). Principal investigator experience was also not associated with a higher total number of protocol deviations or a higher rate of protocol deviations (*p* = 0.7837 and 0.4427, respectively). There was no association found between a higher number of investigators and a higher number of protocol deviations (*p* = 0.1612) or a higher number of coordinators and a higher protocol deviation rate (*p* = 0.7763).

**Fig. 5:**
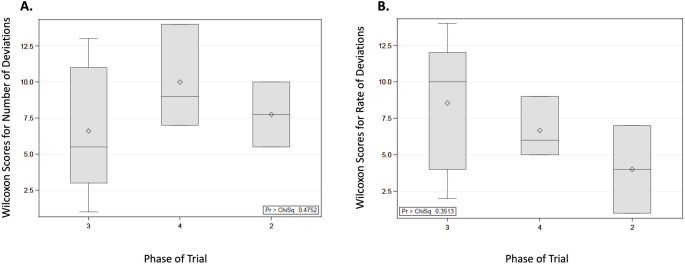
Wilcoxon score distributions for the total number of study protocol deviations based on phase of the trial (**A**) and Wilcoxon score distributions for the rate of study protocol deviations based on phase of the trials (**B**)

**Fig. 6:**
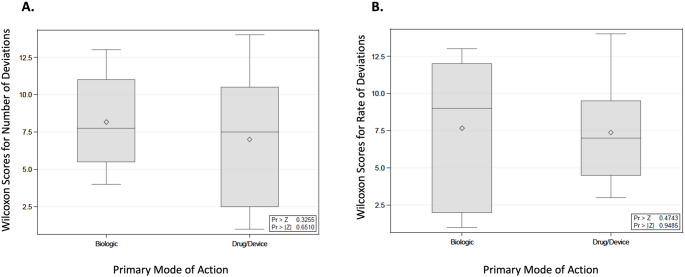
Wilcoxon score distributions for the total number of study protocol deviations based on primary mode of action of investigational product (PMOA) (**A**) and Wilcoxon score distributions for the rate of study protocol deviations based on PMOA (**B**)

The phase 2 trials had a median value of 28 protocol deviations, phase 3 had a median of 12 protocol deviations, and phase 4 had a median of 20 protocol deviations. However, no significant association was observed between the clinical trial phase and the total number of protocol deviations according to the Kruskal-Wallis test, shown in Fig. 5A (*p* = 0.4752). As summarized in Fig. 5B, there was also no association found between clinical trial phase and protocol deviation rates (*p* = 0.3513), with median values of 1.87 protocol deviations rate in phase 2 trials, 3.86 protocol deviations rate in phase 3 trials, and 2.22 protocol deviations rate in phase 4 trials. As shown in Fig. 6A, the Kruskal-Wallis test found no association between the primary mode of action and the number of protocol deviations (*p* = 0.6510). Finally, there was no association observed between the primary mode of action and the rate of protocol deviations, as shown in Fig. 6B (*p* = 0.9485).

## Discussion

ICH GCP guideline for industry E3 Questions & Answers (R1) acknowledges two types of protocol deviations: major (also called important) and minor deviations. It defines an important deviation as the following: “important protocol deviations are a subset of protocol deviations that may significantly impact the completeness, accuracy, and/or reliability of the study data or that may significantly affect a subject’s rights, safety, or well-being.” [18] Examples of major deviation were highlighted as deviations that affect eligibility criteria, and patient safety, or that have a considerable impact on data integrity [18]. 

As shown in Table 2, the small number of major deviations observed in the trial conducted reflects how well our site prioritized the informed consent process, eligibility criteria, and followed the IRB safety reporting guidelines to avoid major deviations. Specifically, 3 major deviations in diabetic foot ulcers (DFU) studies were related to eligibility criteria, 4 deviations related to prohibited concomitant medication being used by the subjects in both venous leg ulcer (VLU) and diabetic foot ulcer (DFU trials); and there were no deviations detected as related to informed consent process or safety events reporting. Noteworthy, all out of 5 deviations observed in category “deviation related to the test article”, 3 did not increase risk to the study subjects and/or affected efficacy and safety in the conducted trials, and therefore, were not considered major deviations upon review by the trial monitors, sponsors and IRBs. However, 2 deviations in this category were deemed as major deviations related to one of the constituent parts of combination product: one of them resulted in lack of efficacy due to wrongful preparation of investigational products and another trigged an adverse event, which required intervention. Overall, 9 (1.34%) of the 671 reported protocol deviations were classified as major deviations.

**Table 2:** Total number of protocol deviations per specific category for all studies analyzed. Typically, categories labeled with asterisks (*) are considered as major study protocol deviations or violations, but it can vary depending on a study protocol. *Abbreviations*: PD- protocol deviations; VLU-venous leg ulcer, DFU- diabetic foot ulcer, SUG- surgical study

Category of Protocol Deviation	Total Number of PDs per category		
	VLU studies	DFU studies	SUG Studies
Study Visit Out of Protocol Window	115	20	45
Missed Study Visit	26	47	62
*Prohibited Concomitant Medication Used by Subject	2	2	0
*Deviation Related to the Test Article	2	3	0
Study Procedure Not Performed or Performed Late	78	31	119
*Eligibility Criteria Not Met	0	3	0
Other	52	14	50
**Sub-Total per category**:	275	120	276
**Total Overall**:	671		

Protocol deviations can arise from patient challenges or study team/site challenges. To minimize the number that stems from study team errors, it is important to ensure proper protocol training at the beginning of the trial and when protocol amendments are introduced [19]. In addition, each coordinator and investigator who is added after the trial start date needs to be trained separately to avoid protocol deviations that result from a lack of knowledge or familiarity with the protocol, site regulations, or reporting guidelines [19]. Given that there was no association found between protocol deviations and the number or experience of study staff, this means that our site was able to effectively train and delegate tasks to various study team members. Additionally, the finding that no association was observed between the number of protocol amendments or amendments that triggered informed consent form (ICF) changes and protocol deviations further demonstrates the ability of our site to effectively manage changes and mitigate risks involved with protocol/ICF changes by implementing timely training of personnel.

Other protocol deviations occur for patient-specific reasons. Some examples include patient noncompliance, scheduling difficulties, or financial issues. This relates to social determinants of health because patients facing certain barriers to general healthcare access might also face challenges to study participation and protocol adherence. Because there were no associations found between protocol deviations and the distribution of gender, age, race, or primary language, this highlights our site’s effort to prevent protocol deviations that result from sociodemographic factor-related challenges. Similarly, the lack of an association between deviations and travel distance, comorbidities, polypharmacy, and BMI illustrates our site’s ability to decrease the prevalence of PDs even for patients who must travel further for study visits or who have a higher disease burden. While no association was observed between median household income and protocol deviations, more deviations were reported for patients who belonged to the uninsured group than for those in the private insurance or government/state insurance groups. However, the relationship was not statistically significant. Overall, the results show that our site can effectively minimize protocol deviations that come from various social determinants of health (SDOH), allowing us to continue to push for the inclusion of diverse patient populations in clinical trials.

We assumed that fewer protocol deviations would occur in trials that were shorter in duration, and study protocol was less complex. Our results showed that there was no correlation between higher complexity scores and a higher number of protocol deviations, proving that even with more difficult trials our site was able to adhere to study guidelines. However, there was a weak correlation and association found between longer study participation and a higher number of protocol deviations. This again emphasizes the importance of the informed consent process, ensuring that patients understand the requirements associated with study participation, including the number of study visits and assessments, before enrollment.

Finally, the reported protocol deviations were broken down into seven categories to pinpoint which aspects of study protocols lead to deviations more often than others. All 14 clinical trials belonged to one of the following three groups: venous leg ulcer (VLU), diabetic foot ulcer (DFU), or surgical (SUG) clinical trials. Table 2 shows the distribution of deviations into the 7 descriptive categories across each type of trial. The category with the highest number of protocol deviations was “Study procedure not performed or performed late,” with 228, or 33.97% of the total number of deviations recorded. Additionally, surgical clinical trials had the most deviations observed (PD total = 276; which most of them 119 observed in category “study Procedure not performed or performed late”, followed by VLU trials with a total of 275, and then DFU trials with a total of 120 deviations (Table 2). By elucidating further which aspects of clinical trials produce the most protocol deviations, we can develop risk-mitigation strategies that target these specific problem areas, and therefore, ensure more accurate and reliable clinical trial data.

Additionally, only 9 (1.34%) of the 671 reported protocol deviations as shown in Table 2 were classified as major deviations or protocol violations (e.g. eligibility criteria non-compliance), either affecting patient safety or data integrity. Regardless of this small percentage, more work can be done by sites to decrease the occurrence of major deviations in clinical trials with combination products. Besides, large numbers of minor study protocol deviations can affect clinical trials data integrity and require effective corrective and preventive actions to mitigate risks to the study participants and preserve data integrity. These findings warrant larger studies for quality contract in trials with combination products need to be conducted in different hospital settings (e.g. private practices, academic hospitals, etc.) to inform development of efficient quality management system that would decreases study protocol noncompliance and mitigate patient’s risk when participating in trials with combination products.

## Limitations

We have performed a retrospective analysis on how study protocol amendments, study personnel and social determinants of health impact clinical trials conduct with combination products at a single clinical research site, affiliated with a safety net hospital with some characteristics and practices that can be specific to our clinical care and clinical research practices. Therefore, large, multi-center, prospective studies in different settings (e.g. academic medical centers, rural clinics, private practices, etc.), with more diverse patient populations, and/or research practices are needed in order to further examine observed correlations/trends, improve quality and data integrity in clinical trials with combination products.

## Conclusion

The finding that longer study participation was found to be associated with a higher number of protocol deviations highlights the importance of the informed consent process, patent compliance, and site preparedness. It is crucial for study staff to effectively communicate what is required of a study patient before they sign a consent form, to ensure that the patient is able to receive the study treatment and remain enrolled for the entire follow up period. Investigational sites can better prepare for patient visits by scheduling study-related labs, assessments, and appointments ahead of time, and by providing patients with reminders for upcoming visits. Despite the result that a larger number of study participants was associated with a larger number of protocol deviations, the rate of deviations was not. This shows that our site was able to balance a larger study population without affecting protocol adherence.

## Data Availability

No datasets were generated or analysed during the current study.
